# Evaluation of the Xpert Carba-R (Cepheid) Assay Using Contrived Bronchial Specimens from Patients with Suspicion of Ventilator-Associated Pneumonia for the Detection of Prevalent Carbapenemases

**DOI:** 10.1371/journal.pone.0168473

**Published:** 2016-12-16

**Authors:** Almudena Burillo, Mercedes Marín, Emilia Cercenado, Guillermo Ruiz-Carrascoso, María Jesús Pérez-Granda, Jesús Oteo, Emilio Bouza

**Affiliations:** 1 Clinical Microbiology and Infectious Diseases Department, Hospital General Universitario Gregorio Marañón, Madrid, Spain; 2 Instituto de Investigación Sanitaria Gregorio Marañón, Madrid, Spain; 3 Medicine Department, School of Medicine, Universidad Complutense de Madrid, Madrid, Spain; 4 CIBER de Enfermedades Respiratorias (CIBERES CB06/06/0058), Madrid, Spain; 5 Microbiology Department, Hospital Universitario La Paz, Madrid, Spain; 6 Department of Anesthesiology, Cardiac Surgery Intensive Care Unit, Hospital General Universitario Gregorio Marañón, Madrid, Spain; 7 Antibiotic Laboratory, Bacteriology Department, Centro Nacional de Microbiología, Instituto de Salud Carlos III, Majadahonda, Spain; Cambridge University, UNITED KINGDOM

## Abstract

There is a critical need for rapid diagnostic methods for multidrug-resistant (MDR) pathogens in patients with a suspicion of ventilator-associated pneumonia (VAP). The Xpert Carba-R detects 5 targets for carbapenemase-producing organisms (bla_KPC_, bla_NDM_, bla_VIM_, bla_OXA-48_, and bla_IMP-1_). Our objective was to evaluate the performance of this assay directly on bronchial aspirates and to correlate the cycle number for a positive result (Ct) with the bacterial count. Bronchial aspirates from patients with a suspicion of VAP were spiked with a dilution of 1 of 4 MDR organisms carrying the resistance genes detected by the test prepared to a final concentration of 10^2^−10^5^ cfu/mL. We used a ROC curve and provided areas under the curve (AUC) with their 95% confidence intervals (CI). A point of maximum sensitivity (Se) and specificity (Sp) was derived and validity indices were calculated. One hundred contrived tests were performed. Se and Sp were 100% for all bacterial counts. A positive sample with a Ct ≤24.7 corresponded to a count ≥10^5^ cfu/mL; if the Ct was within the range >24.7-≤26.9, this corresponded to a count ≥10^4^ cfu/mL. When the Ct was >26.9, this corresponded to a count <10^4^ cfu/mL. The Xpert Carba-R detects carbapenemase-producing organisms directly in contrived bronchial aspirates. Still, an important issue to consider is that the number of gene copies may vary according to many factors in vivo. If confirmed in further studies, the strong correlation observed between Ct values and the results of semiquantitative cultures suggests this test could serve to differentiate between infection and colonization in routine clinical practice.

## Introduction

Hospital-acquired pneumonia (HAP), particularly ventilator-associated pneumonia (VAP), is one of the leading causes of infection and death in the healthcare setting [1–5]. Its incorrect or delayed treatment in the first few hours gives rise to a worse prognosis and a higher mortality rate [6–8]. Inappropriate antibiotics are also a cause of adverse events and unnecessary expenses [9]. Therefore, the aetiologic diagnosis of VAP is a microbiological emergency because of its impact on the morbidity and mortality of this disease.

At present, multidrug-resistant (MDR) pathogens are emerging among both Gram positive organisms (e.g., MRSA) and Gram negative organisms (e.g., carbapenem-resistant enterobacteriaceae -CRE-, *Pseudomonas aeruginosa*, and *Acinetobacter* spp)., due to acquisition of a variety of resistance mechanisms [10]. This resistance compromises effective treatment.

Generally, treatment of VAP caused by one of these organisms is of considerable concern to the clinician because therapeutic options are limited by resistance [11]. Therefore, there is a critical need for rapid diagnostic methods for these pathogens and to exclude the presence of worrisome mechanisms of resistance [12,13].

PCR-based methods that detect carbapenem resistance genes have recently been developed [14,15]. These tests have proved to be extremely sensitive and specific in the detection of colonization in rectal swabs.

Our hypothesis was that one of these assays, the Xpert Carba-R, which detects 5 targets for carbapenemase-producing organisms (bla_KPC_, bla_NDM_, bla_VIM_, bla_OXA-48_, and bla_IMP-1_) may be used on lower respiratory tract (LRT) samples obtained in mechanically-ventilated patients to detect carbapenemase-producing organisms rapidly (within one hour) with a comparable accuracy to traditional susceptibility testing methods. This assay is FDA approved and has received the CE mark approval for use on rectal swabs. The result for each target is determined separately. Our group has already showed the usefulness of the Xpert MRSA/SA SSTI assay for the detection of the presence of MRSA and MSSA in a complex sample, such as bronchial aspirates, of patients with a suspicion of VAP [16].

The aim of this study was to evaluate the performance of Xpert Carba-R directly on LRT samples compared with the reference method, i.e., semiquantitative culture.

## Material and methods

### Setting

Our institution is a 1550-bed tertiary university hospital,serving a population of approximately 750,000 inhabitants in Madrid, Spain. It has 3 different ICUs for adult patients (medical ICU, general postsurgical ICU, and cardiac surgery ICU) with a total of 42 beds.

### Study period and clinical samples

The samples used were leftover LRT samples no more than two days old from patients admitted to the ICU with a suspected respiratory tract infection acquired during mechanical ventilation. Cultures of these samples turned negative. Specifically, it was checked that these samples lacked organisms carrying genes coding for carbapenemase production (bla_KPC_, bla_NDM_, bla_VIM_, bla_OXA-48_, and bla_IMP-1_).

### Microbiologic management of samples for direct Xpert Carba-R testing

Adequate mixing of the respiratory sample is essential when inoculating lower respiratory tract specimens from patients, with a predetermined concentration of a microorganism. It is necessary to obtain a homogenous bacterial mixture so that the cycle number in which the sample becomes positive may be correlated with the bacterial count of the sample.

Samples of bronchial aspirates were homogenized by vortexing at 3,000 rpm for one minute, using the sample reagent of the Xpert Carba-R assay (vol:vol). Each sample was then divided in four aliquots and these were spiked with a dilution of one of four MDR organisms carrying a single resistance mechanism detected by the test, prepared in sterile water to a final concentration of 10^2^, 10^3^, 10^4^ and 10^5^ cfu/mL. We used the following organisms: a strain of a KPC-2 producing *Citrobacter freundii*, a strain of a VIM-1 producing *Enterobacter cloacae*, a strain of OXA-48 producing *Klebsiella pneumoniae*, and a strain of NDM-7 producing *Klebsiella pneumoniae*. These organisms were identified using MALDI-TOF (Bruker Daltonics, Bremen, Germany). When our study was conducted, no IMP1-1 carbapenemase had been detected in our country, so this target was not investigated. The presence of genes encoding carbapenemases, bla_KPC_, bla_VIM_, bla_OXA-48_ and bla_NDM_, was confirmed by PCR amplification with specific primers and DNA sequencing [17–20]. Each prepared sample was inoculated on three blood agar plates to determine bacterial counts, like in the standard semiquantitative culture method of LRT samples [21]. Colonies were counted after 48 h of incubation at 37°C in ambient air.

Following inoculation of the organisms into the sample reagent, 1.7 mL of the sample reagent were transferred into the cartridge. The assay was run on the GeneXpert platform per the manufacturer’s instructions. As a negative control, a LRT sample without the addition of any carbapenemase producing organisms was used.

We evaluated the correlation between the cycle number at which the sample fluorescence crossed above the established cutoff for diagnosis (Ct, crossing-point or threshold cycle) and the final plate counts, in an attempt to differentiate between colonization and infection.

We used a receiving operating curve (ROC) and provide areas under the curve (AUC) with their 95% confidence intervals. From these AUCs, a point of maximum sensitivity (Se) and specificity (Sp) was derived and validity indices (sensitivity, specificity, positive (PPV) and negative predictive values (NPV), and positive (PLR) and negative likelihood ratios (NLR), with their 95% confidence intervals) calculated. If the PLR is over 5 and the NLR is under 0.10, it may be concluded that the test is essential for a diagnosis and of high validity for use in routine clinical practice. The software packages used were SPSS ver. 15.0 and Epidat ver. 3.1.

### Sample size

Twenty consecutive bronchial aspirates without organisms carrying the genes coding for carbapenemase production were included. Each aspirate was spiked with an organism containing a single resistance mechanism prepared to four final concentrations (10^2^, 10^3^, 10^4^ and 10^5^ cfu/mL). Twenty negative controls were included. Therefore, a total of one hundred Xpert Carba-R tests were performed.

### Ethics statement

The Institutional Review Board of the Instituto de Investigación of the Hospital General Universitario Gregorio Marañon waived the need for a written informed consent from patients, as included patients were not subject to extra procedures or questions. Samples were collected as part of standard care. The project number was MICR-12-01 and the number of the authorization was 179/14. This was a purely based laboratory intervention without involvement of patients. Samples employed in the analyses were de-identified before access.

## Results and Discussion

Of the hundred contrived tests that were performed, there were no false positive or false negative results, at any dilution used. The sensitivity and specificity of the test were 100% (95% CI 95.5–100%); it detected all carbapenemase-producing organisms at all concentrations studied, and there were no false positive results with any of the negative controls.

There was one mechanical error due to the characteristics of the sample, in one instance one fluorescence check value was below the expected minimum and the test was aborted, and in another instance an ultrasonic failure occurred and the DNA could not be extracted. There were no invalid results.

The correlation between the cycle number at which the sample fluorescence crossed above the established cutoff for diagnosis (crossing-point or threshold cycle) and the final count of organisms is shown in Table 1.

**Table 1:** Performance of the Xpert Carba-R in lower respiratory tract samples spiked with carbapenemase-producing organisms. Correlation between the crossing-point or threshold cycle and the final count of organisms

Bacterial count	AUC	95% CI	Ct	Sensitivity	Specificity
**10**^**5**^ **vs. 10**^**2**^**−10**^**4**^ **cfu/mL**	0.97	0.93–1.00	≤24.75	95%	95%
**10**^**5**^ **vs. 10**^**4**^ **cfu/mL**	0.91	0.79–1.00	≤24.75	95%	84%
**10**^**5**^ **vs. 10**^**2**^**−10**^**3**^ **cfu/mL**	1.00	1.00–1.00	≤26.95	100%	100%
**10**^**4**^ **vs. 10**^**2**^**−10**^**3**^ **cfu/mL**	0.97	0.92–1.00	≤27.10	84%	100%

Cfu: colony forming units; AUC: area under the curve; CI: confidence interval; Ct: crossing point or threshold cycle

When we used a crossing-point or threshold cycle ≤24.7 to differentiate between samples with a bacterial count of 10^5^ cfu/mL, compared to the rest of the bacterial counts, we obtained the following validity indices: Se 94.7 (82.1–100.0), Sp 94.7 (88.1–100.0), PPV 85.7 (68.4–100.0), NPV 98.2 (93.7–100.0), PLR 18.00 (5.95–54.43), NLR 0.06 (0.01–0.37) (Table 2).

**Table 2:** Crossing-point or threshold cycle used to differentiate between infection and colonization

Bacterial count	Crossing point	Se (95%CI)	Sp (95%CI)	PPV (95%CI)	NPV (95%CI)	PLR (95%CI)	NLR (95%CI)
**≥10**^**5**^ **cfu/mL**	≤24.7	94.7 (82.1–100.0)	94.7 (88.1–100.0)	85.7 (68.4–100.0)	98.2 (93.7–100.0)	18.00 (5.95–54.43)	0.06 (0.01–0.37)
**<10**^**4**^ **cfu/mL**	>26.9	84.2 (65.2–100.0)	100.0 (98.7–100.0)	100.0 (96.9–100.0)	92.7 (83.5–100.0)	Undefined	0.16 (0.06–0.45)

Se: sensitivity; Sp: Specificity; PPV: positive predictive value; NPV: negative predictive value; PLR: positive likelihood ratio; NLR: negative likelihood ratio; cfu: colony forming units.

When we used a crossing-point or threshold cycle ≤26.9 to differentiate between samples with a bacterial count of 10^4^ cfu/mL, compared to samples with bacterial counts of 10^2^−10^3^ cfu/mL, we obtained the following validity indices: Se 84.2 (65.2–100.0), Sp 100.0 (98.7–100.0), PPV 100.0 (96.9–100.0), NPV 92.7 (83.5–100.0), PLR undefined (since there were no false positive results), and NLR 0.16 (0.68–1.01) (Table 2).

Therefore, from our results we can conclude that if a sample is positive with a Ct ≤24.7, this corresponds to a bacterial count ≥10^5^ cfu/mL indicative of infection [22–24]. If the Ct is within the range >24.7-≤26.9, this corresponds to a bacterial count ≥10^4^ cfu/mL. And when the Ct is >26.9, this corresponds to a bacterial count <10^4^ cfu/mL, suggesting colonization.

There were no differences in AUC by type of carbapenemase, but we must take into account the limited sample size in this analysis.

We here describe the successful use of a commercial diagnostic test, designed to identify patients showing gastrointestinal colonization with carbapenemase-producing organisms, to detect bacterial carbapenem resistance genes in lower respiratory tract samples. Our results could be used as proof-of-concept data for validation of this assay for this indication.

To validate diagnostic platforms, the ideal would be to use well-characterized LRT samples from a range of clinical settings, since this will enable assessment of a wide panel of pathogens. However, this involves many technical and regulatory hurdles. In the meantime, despite several limitations, spiked LRT samples seemed most appropriate for our research study [25].

The test's performance even using these complex samples was excellent. The sample reagent allows the organisms to be freed from mucus and other respiratory secretions resulting in liquefied and homogenized sample that is easy to manipulate.

Using the criteria that we established, the test was also able to differentiate between bacterial counts indicative of infection or colonization in routine clinical practice. The capacity of this test to quantify organisms has been also reported by Stenehjem et al., who determined nasal colonization bioburdens in swabs using the Xpert MRSA test [26]. The most obvious benefit of this test is that it provides information on which to base the choice of antibiotic treatment for patients with suspected pneumonia in less than an hour after the sample arrives at the laboratory. A similar test, the Cepheid Xpert^TM^ *van*A/*van*B, has already proved to be cost-effective for the rapid detection (in approximately one hour) of glycopeptide-resistant enterococci in rectal swabs of in-patients [27].

An additional benefit is that these tests enable rapid decisions regarding the best infection control strategy to be made, with a minimal economic loss in terms of admissions and transfers [27].

Resistance to antibiotics threatens our health. As pointed out by Chambers et al., resistance is a complex, multifactorial, unavoidable problem that is, however, potentially manageable [28]. The Antibacterial Resistance Leadership Group (ARLG) of the United States National Institute of Allergy and Infectious Diseases (NIAID) of the National Institutes of Health (NIH) has launched a program to address this issue, which includes the use of simple point-of-care diagnostics to detect drug resistance and guide treatment.

One of the limitations of the Xpert Carba-R test is that the amount of DNA detected reflected by the Ct value may be conditioned by the presence of multiple gene copies. Yet, this possibility, which has not been extensively studied yet, is infrequent [29]. In addition, the possible presence in patient samples of DNA in dead cells could interfere with test results if performed before DNA degradation takes place. Nevertheless, in one study that evaluated whether rectal swabs are suitable for quantifying the carriage load of KPC-producing carbapenem-resistant *Enterobacteriaceae*, the authors found that the results of a quantitative PCR assay performed directly on rectal swabs showed a higher correlation than the quantitative culture-based method with the reference method (concentration of 16S rRNA genes), and that this quantitative PCR was suitable for quantifying these microorganisms [30]. In summary, our results need to be considered with caution and the strong correlation detected between Ct values and bacterial counts would need to be confirmed in clinical samples.

If indeed our results are confirmed, the next step would be to validate the test for use on samples from patients with a suspected infection caused by these organisms. We would also need to examine the direct impacts of the use of this test on clinical factors (e.g., days of fever), therapeutic factors (e.g., prescription time, adequacy of antibacterial treatment, treatment adjustment, treatment duration, antibiotic consumption), outcome measures (e.g., days on mechanical ventilation, days of ICU stay, days of hospital stay, *Clostridium difficile*-associated diarrhea, mortality attributed to pneumonia, all-cause hospital mortality) and economic factors (e.g., direct antibiotic costs per pneumonia episode) [31].

## Conclusions

In conclusion, the Xpert Carba-R detects the resistance genes of carbapenemase-producing organisms when directly used on bronchial aspirates. If confirmed in clinical samples, the correlation detected between Ct and semiquantitative culture results could serve to differentiate between infection and colonization in routine clinical practice.
